# Shexiang Baoxin Pill, a Proprietary Multi-Constituent Chinese Medicine, Prevents Locomotor and Cognitive Impairment Caused by Brain Ischemia and Reperfusion Injury in Rats: A Potential Therapy for Neuropsychiatric Sequelae of Stroke

**DOI:** 10.3389/fphar.2021.665456

**Published:** 2021-04-27

**Authors:** Zong-Shi Qin, Yu Zheng, Xi-Dan Zhou, Dong-Dong Shi, Dan Cheng, Chun Shum Shek, Chang-Sen Zhan, Zhang-Jin Zhang

**Affiliations:** ^1^ School of Chinese Medicine, LKS Faculty of Medicine, The University of Hong Kong, Hong Kong, China; ^2^ The Brain Cognition and Brain Disease Institute, Shenzhen Institutes of Advanced Technology, Chinese Academy of Sciences, Shenzhen, China; ^3^ Shanghai Mental Health Center, School of Medicine, Shanghai Jiao Tong University, Shanghai, China; ^4^ Shanghai Hutchison Pharmaceuticals Ltd., Shanghai, China; ^5^ Shanghai Engineering Research Center for Innovation of Solid Preparation of TCM, Shanghai, China

**Keywords:** middle cerebral artery occlusion, locomotor ability, post stroke cognitive impairment, shexiang baoxin pill, ischemic stroke

## Abstract

Ischemic stroke is a common type of cerebrovascular event and also the leading cause of disability. Post-stroke cognitive impairment occurs frequently in stroke survivors. Shexiang Baoxin Pill (SBP) is a proprietary Chinese medicine, initially used to treat cardiovascular diseases. Herein, we aim to explore the effects of SBP on oxygen glucose deprivation and reoxygenation (OGD/R) in neuronal cells (CATH.a) and cerebral ischemia/reperfusion injury induced post-stroke cognitive impairment in middle cerebral artery occlusion (MCAO) rat model. MCAO rats received two doses of oral SBP treatment (28 or 56 mg/kg) after 1 h of operation and once daily for 2 weeks continuously. Behavioral tests, immunoblotting, and immunofluorescence were examined after 14 days. Current data suggest that SBP enhanced cell viability and downregulated apoptosis via activating the PI3K/Akt signaling pathway in CATH. a cells. Furthermore, 14 days of SBP treatment promoted the recovery of learning and locomotor function in the MCAO rats. SBP up-regulated the expression of p-Akt, p-GSK3β, as well as the expression of NMDAR1, PSD-95, and AMPAR. Also, SBP down-regulated the expression of p-CaMKII. These results indicated that long-term SBP treatment might be a potential option for cognitive impairment induced by the ischemic stroke.

## Introduction

Stroke is a leading cause of death and disability. Of all strokes, 87% are ischemic stroke (G.B.D.N. Collaborators, 2019a; G.B.D.S. Collaborators, 2019b; Zhou et al., 2019a). In the ischemic region, neurons rapidly die due to a cascade of physiological changes. With the development of critical medicine, the death rate of stroke has been significantly decreased. It has been confirmed that stroke could result in cognitive impairment also known as post-stroke cognitive impairment (Pasi et al., 2012; Shih et al., 2013). However, covered by severe physical disability, post-stroke cognitive impairment is likely to be ignored in most cases (Pasi et al., 2012; Sun et al., 2014; Kwon et al., 2020). Delayed cellular loss within the hippocampus correlated with cognitive deficits and long-term prognosis following experimental or clinical ischemic stroke.

It has been known that ischemic stroke is often accompanied by neuronal damage and apoptosis, and activated phosphatidylinositol 3-kinase (PI3K) plays a key role in the inhibition of programmed cell death (Uzdensky, 2019). As an important candidate to regulate the cell-survival responses attributed to PI3K, protein kinase B (Akt) has been implicated as an anti-apoptotic in the cell death paradigm during ischemic stroke. Previous studies have demonstrated the role of Akt in apoptosis suppression (Lv et al., 2017). Meanwhile, it has been realized that the recovery of neurological function after stroke is associated with brain plasticity (Dimyan and Cohen, 2011). The initiation of apoptosis could be inhibited by the increasd synapses, which could also enhance the activity of neurons in the ischemic region of brain. In the excitatory synapses, postsynaptic density protein 95 (PSD95), a member of the PSD family, could bind to the NMDA receptor and neurotransmitter nitric oxide synthase to organize a protein complex, which plays an important role in the excitatory balance of synapses via regulating the conduction of excitatory signaling (Keith and El-Husseini, 2008).

Traditional Chinese medicine (TCM) has been used for ischemic stroke and recovery from the sequelae of stroke for a long time. Shexiang Baoxin Pill (SBP) is a well-known composite TCM formulae in China, which has been approved by the Chinese Food and Drug Administration and used in clinical practice. It is widely used in stable angina pectoris and chest pain, caused by coronary heart disease (Yan et al., 2006; Zhang et al., 2017a; Dong et al., 2018; Lu et al., 2018). Pharmacological evidence indicates that SBP reduces the infarct volume of the heart, anti-inflammation, and promotes angiogenesis (Jiang et al., 2011; Xiang et al., 2012; Xiang et al., 2013). A recent study illustrated that SBP might be effective in treating neurodegenerative diseases (Xu et al., 2019). Meanwhile, increasing pharmacological studies demonstrated that the main components of SBP such as muscone, ginsenosides, bile acid, and taurocholic acid could pass through rat BBB, distributing into the brain (Wang et al., 2014; Huang et al., 2015; Shen et al., 2016). Besides, synthetic borneal could improve the drug delivery to the brain, these findings suggest that SBP might have potential application in neurological disorders (Zhang et al., 2017b). As the representative components of *Panax ginseng*, ginsenoside Rb1 and Re have been reported to reduce acute brain lesions, neurological deficits score, and ameliorate ischemia-induced place navigation disability in MCAO rats (Zhou et al., 2006; Zhu et al., 2012; Zhou et al., 2014). Muscone and cinnamic acid also have neuroprotective effects in MCAO rats (Wei et al., 2012; Du et al., 2018; Shen et al., 2020). Because of the characteristics of Chinese medicine, multi-component could act on multiple targets and pathways (Lu et al., 2018; Lu et al., 2019). Several *in vitro* and *in vivo* studies explored the potential roles of SBP in treating neurological disorders (Dong et al., 2018; Zhou et al., 2019b). SBP reverses the stress-suppressed levels of neurotransmitters’ metabolites and neurotrophic factors in the brain of chronic stress-induced depression mice (Zhou et al., 2019a). The therapeutic effects of SBP might be related to the properties of SBP in cytoprotection and immunomodulation (Zhou et al., 2016; Zhang et al., 2017a). As a multi-component Chinese medicine, SBP may achieve its therapeutic efficacy via active components that regulate molecular networks (Jiang et al., 2009; Zhou et al., 2019b) (Table 1). In the present study, we tested the effect of SBP for consequently cognitive impairment induced by ischemic stroke.

**TABLE 1:** Individual medicinal materials of the SBP.

Material name	Ratio (%)	Full scientific name	Major pharmacologically active constituents
Artificial mouchus	6	The dried preputial secretion of *Moschus berezovskii Flerov, Moschus sifanicus Przewalski,* or *Moschus moschiferus Linnaeus*	Muscone, testosterone
Calculus bovis artifactus	27	*Panax ginseng* C.A. Mey., root	Cholic acid, deoxycholic acid, ursodeoxycholic acid, chenodeoxycholic acid, bilirubin, cholesterol
Radix Ginseng	24	*Cinnamomum cassia* (L.) J. Presl., bark	Ginsenoside Ra1/2, Ginsenoside Rb1/2/3, Ginsenoside Re
Venenum bufonis	4	The dried secretion of *Bufo gargarizans Cantor* or *Bufo melanostictus Schneider*	Cinobufagin, resibufogenin, resibufagin, gamabufotalin, bufalin, 1β-hydroxylbufalin, arenobufagin, bufotalin, telocinobufagin, telibufagin
Cortex cinnamomi	8	*Liquidambar orientalis* mill., resin	Cinnamic acid, cinnamaldehyde
Styrax	4	The dried gall-stone of *Bos taurus domesticus Gmelin*	Benzyl benzoate
Borneolum syntheticum	19	*Borneolum Syntheticum* or *Dryobalanops aromatica* C.F. Gaertn, resin	Borneol, isoborneol

## Methods

### UHPLC Analysis

SBP (manufacturer batch number: 160,504) was kindly provided by Shanghai Hutchison Pharmaceuticals Company (Shanghai, China). SBP contains 7 materia medicas or extracts, including Moschus, Radix Ginseng (*Panax ginseng C.A.Mey., root*), Styrax (*Liquidambar orientalis Mill., resin*), Cinnamomi Cortex (*Cinnamomum cassia (L.) J. Presl., bark*), Bufonis Venenum, Bovis Calculus Artifactus and Borenolum Syntheticum (*Dryobalanops aromatica C.F.Gaertn, resin*). Supplementary Figure S1 summarized medicinal materials of SBP. Liquid chromatography system (UHPLC, Thermo Fisher Scientific, United States) equipped with a C18 column (2.1 × 150 mm, Thermo Fisher Scientific, United States) as stationary phase was used to analyze and separate the sample components of SBP. The column temperature was maintained at 25°C, injection volume was 5 μL and the flow rate was 0.3 ml/min. Mobile phases A and B were acetonitrile and 0.1% H_3_PO_4_ in water correspondingly. The gradient elution profile was as follows: 0–3 min, 98% B; 3–6 min, 85% B; 6–18 min, 73% B; 18–28 min, 71% B; 28–38 min, 67%B; 38–45 min, 65% B; 45–52 min, 35% B; 52–60 min, 5% B.

### Cell Culture

CATH.a cell line was established from cultures of a tumor that arose in the brain of a transgenic mouse, which was obtained from the American Type Culture Collection (ATCC, VI, United States). Cells were cultured with RPMI-1640 medium (ATCC, VI, United States) containing 2 mM ,L-glutamine, 10 mM sodium pyruvate, 4,500 mg/L glucose, 1500 mg/L sodium bicarbonate, 8% horse serum (HS), 4% fetal bovine serum (FBS), and 1% penicillin and streptomycin (PS) with a humidified atmospheric air (78% N_2_ and 21% O_2_) supplied with 5% CO_2_.

### Oxygen Glucose Deprivation and Reoxygenation and Shexiang Baoxin Pill Treatment

To induce the OGD/R model, we firstly cultured CATH. a cells were with glucose-free 1640 medium supplemented with 4% HS and 1% FBS. The incubator condition was maintained with 95% N_2_ and 5% CO_2_ at 37°C for 4 h and then returned to the humidified CO_2_ incubator perfused with 21% O_2_ mixing 5% CO_2_ for 20 h. SBP co-treatment was administered during the reoxygenation period. In preparing SBP extracts, 50 g powders of SBP was sonicated in 95% ethanol in a proportion of 1:4 (w/v; 200 ml) for 30 min at 37°C. Then the solution was filtered to remove insoluble aggregates and placed onto a rotary evaporator to remove the solvent, after which the solid was put onto a high vacuum apparatus overnight. Finally, the extract of SBP was dissolved in dimethylsulfoxide (DMSO) with a stock solution at 500 mg/ml.

### Cell Viability Assays

The viability of CATH. a was detected by Cell Counting Kit-8 (CCK8) assay (Dojindo, United States). Cells were plated in 96-well plate for 24 h and treated with drugs for 24 h. After treatment, 10 μL per well of CCK8 was added and the plates were incubated at 37°C for 1 h. Then the plate were quantified by measuring absorbance at 495 by multi-plate reader (Model 680, Bio-Red, Laboratories Inc., United States).

### Flow Cytometry

Cell apoptosis was measured by PE Annexin V apoptosis detection kit (BD Biosciences, United States). The cell suspension was incubated at room temperature with 7-AAD and PE Annexin for 15 min in the dark. For each sample, the cells were analyzed using NovoCyte Advanteon BVYG (Agilent, United States).

### Animals

All animal care and experimental protocols were approved by the Committee on the Use of Live Animals in Teaching and Research of the University of Hong Kong (CULATR No. 4840-18). Animal studies are reported in accordance with the ARRIVE guidelines (Nam et al., 2018). Male Sprague-Dawley (SD) rats weighing 270 ± 10 g were used in the experiment. The rats were housed 2 per cage on a 12 h light/dark cycle at temperature (23°C) with water and food available *ad libitum*.

### Middle Cerebral Artery Occlusion Model Operation

The cerebral ischemia/reperfusion MCAO model was conducted according to the previous protocol (Liu and McCullough, 2011; Zheng et al., 2020). Briefly, rats were anesthetized with 4% isoflurane (Abbott, IL, United States) and maintained with 1.5% isoflurane via inhalation. The common carotid artery, external carotid artery (ECA), and internal carotid artery (ICA) were exposed and ligated on the left side. A 0.36-mm monofilament suture (L3600, Jialing Co. Ltd., China) was inserted into the ECA and advanced through the ICA to the ostium to occlude the middle cerebral artery. Sham control rats were subjected to similar operations without occlusion. The suture was removed after 2 h to cause reperfusion. SBP or saline was orally administered to the animal immediately before reperfusion. The reperfusion process continued for 24 h. Rats were allowed free access to food and water after recovery from anesthesia. Animals were excluded from analysis when the following occurred: modified Neurological Severity Score less than 5 at 3 h after ischemia/reperfusion injury, intracerebral hemorrhage, underweight (animal’s body weight loss exceeds 30% pre-surgical weight) or died. Two investigators (YZ and XZ) blinded to the experimental grouping and drug treatment performed the neurobehavioral assessments.

### Shexiang Baoxin Pill Preparation and Treatment

The quality control of SBP was as described in the Chinese Pharmacopoeia. SBP was dissolved in a vehicle containing a 0.5% aqueous solution of sodium carboxymethyl cellulose (CMC) and administered via oral gavage per day. Two doses of SBP (28 mg/kg and 56 mg/kg) were calculated in accordance with the clinical usage of SBP in the Chinese population (Wei, 2010). The solution of SBP or CMC was administered to the rats 1 h after ischemia. A total of 60 Rats were randomly divided into 4 groups: (1) sham operation group (*n* = 15), (2) MCAO group (*n* = 15), (3) SBP 28 mg/kg group (*n* = 15), and (4) SBP 56 mg/kg group (*n* = 15).

### Infarct Size Measurement

To measure the infarct size of the brain, rats were transcardially perfused with PBS under anesthesia (90 mg/kg ketamine plus 10 mg/kg xylazine) to collect brain tissues. The isolated brain tissues were sectioned into 2 mm slices and stained in 2% TTC (Sigma-Aldrich, MO, United States) solution for 15 min in a dark place. Infarction size was quantified by measuring the white infarcted area and reddish-purple non-infarcted area using ImageJ software (National Institutes of Health, United States). Percentage of infarct size was calculated referring to the following equation (McBride et al., 2015):

$$\text{Infarct size}\left(\text{\%}\right)=\left\{\frac{\left[\sum_{i}\left(C_{i}-N_{i}\right)\right]}{\sum_{i}C_{i}}\right\}\times 100\%$$ (1)

In which, *C*_*i*_ represents the area of the contralesional hemisphere and *N*_*i*_ represents the area of the nonischemic tissue of the ipsilesional hemisphere.

### Morris Water Maze Test

The Morris Water Maze test was performed to assess the spatial memory of post-stroke rats. The test was conducted according to the previous protocol (Zheng et al., 2020). From day 9 after MCAO, the rats were trained to find a hidden hyaline platform below the water. Each rat underwent 2 training trials per day for 5 continuous days (from day 9 to day 13), with 30 min intervals between trials. For rats that could not find the platform within 60 s were guided to the platform by the researcher. The time to the platform was recorded for each training trial. A probe test was carried out 24 h after the last training trial (day 14). In the probe test, we removed the platform and the rat was allowed to swim within 60 s. The duration spent in and frequency of entering the quadrant where the platform was previously located was recorded.

### Open Field Test

The open field apparatus used the same apparatus as the novel object recognition test at day 13 after MCAO. The testing was conducted in dimly lit conditions without experimenters’ presence. During a 10 min test period, the total distance moved and the time used in target zones were recorded. The apparatus was cleaned with 75% alcohol after each test to eliminate the preorder rat’s odor.

### Locomotor Activity Test

The locomotor activity was tested on days 8 and 15 after MCAO. Briefly, rats were placed on the rotarod cylinder (7750, Ugo Basile, Italy). Each rat was given two training trials (4 rev/min for 5 min) 24 h before the final test. The rolling speed was initially set as 4 rev/min and gradually accelerated to 40 rev/min over 3 min. The duration of the animals stayed on the rotarod was recorded for between-group comparison.

### Novel Object Recognition Test

The novel object recognition test was performed on day 15 after MCAO in a 100 × 100 × 60 cm black plastic quadrangular apparatus. During the training phase (day 14 after MCAO), the rat has encountered two familiar objects (transparent plastic cube) in opposite corners for 10 min. After 24 h, The rat was placed in the same apparatus and presented with a familiar object and a novel object (white plastic cylinder) in the same places as the training phase for 10 min. Exploration is defined as exploring the object at a distance ≤2 cm or touching with its nose. Videotapes were analyzed using the SMART video tracking system (version 3.0, Panlab, Harvard Apparatus, Spain). The total exploring time for both objects was calculated, and the recognition index was calculated and analyzed through the following equation (Antunes and Biala, 2012):

$$\text{Recognition index}\left(\text{\%}\right)=\frac{T_{n}}{T_{t}}\times 100\%$$ (2)

In which, *T*_*n*_ represents the time spent exploring the novel object, *T*_*t*_ represents the total time spent exploring both familiar and novel objects.

### Western Blot

Cells and tissues were extracted radioimmunoprecipitation assay (RIPA) buffer (Sigma-Aldrich, United States) supplemented with 1% protease and phosphatase inhibitor cocktail (Thermo Fisher Scientific, United States). Equal amounts of proteins were separated by 10% SDS-polyacrylamide gel electrophoresis, transferred onto a 0.22-μm polyvinylidene fluoride membrane (PVDF, Bio-Rad Laboratories, United States), and blocked with 5% BSA. The PVDF membrane was incubated with antibodies of p-CaMKII (sc-32289), CaMKII (sc-13141) (1:1,000 for both, Santa Cruz Biotechnology), p-Akt (#9271), Akt (#9272S), p-GSK3β (#9336S), GSK3β (#9315), NMDAR1 (#4204), PSD-95 (#2507S), AMPAR(#13185) (1:1,000 for all, Cell Signaling Technology), and GAPDH (sc-47724) (1:5,000, Santa Cruz Biotechnology) overnight at 4°C. After the chemiluminescence staining (GE Healthcare, IL, United Kingdom) the bands were visualized by ChemiDoc™ XRS + System (Bio-Rad Lacoratories, Inc., CA, United States).

### Immunofluorescent Staining

The rats brain tissues were fixed in 4% PFA for 12 h and then transferred in 30% sucrose solution for 1 week. After dehydration, the brains were positioned for coronal cut using a cryo-microtome (Leica, Germany) at −20°C. The sections were blocked with the blocking buffer (5% BSA/0.3% Triton™ X-100) for 2 h at room temperature. Then the sections were incubated with primary antibodies against NeuN (MAB377) (1:500, Millipore) and PSD-95 (#2507S) (1:500, Cell signaling technology) overnight at 4°C. After that, the sections were incubated in fluorescent-conjugated secondary antibodies (Alexa Flour 488 and 594, Invitrogen, 1:500). At last, the sections were stained with 4ʹ,6-diamidino-2-phenylindole (DAPI, Sigma-Aldrich, MO, United States) and mount with mounting medium (Teomics, United States). The images of immunofluorescent staining were detected using a confocal laser scanning microscope (LSM 880, Carl Zeiss, Germany).

### Data Analysis

All data are presented as means ± standard error (SE). Videotapes of animal behaviour tests were analyzed using the SMART video tracking system (version 3.0, Panlab, Harvard Apparatus, Spain). For the data of the training test during MWM, two-way ANOVA was used to compare each group at different time points. Other measurements were tested using one-way ANOVA. Dunnett’s test was conducted as the post hoc multiple comparison test. All statistical analyses were performed using GraphPad Prism (Version 6.0, GraphPad Software Inc., CA, United States). All statistical tests were two-sided and *p* < 0.05 was considered statistically significant.

## Results

### Quality Assessment of SBP

By comparing the retention time and UV spectrum, we identified that ginsenoside Re, trans-cinnamic acid, ginsenoside Rb1, and cinobutagin of SBP (Supplementary Figure S1). The contents of ginsenoside Re, ginsenoside Rb1, trans-cinnamic acid, and cinobutagin were determined as 438.8, 310.0, 16.0, and 58.0 ug/ml, respectively. As one of the main compounds of SBP, the peak of ginsenoside Re was relatively higher. Quality consistency was measured by calibrating the area of four common peaks in the chromatograms of three different batches of SBP, and the result showed that the chemical composition of SBP was consistent within batches of production.

### Shexiang Baoxin Pill Enhanced Cell Viability and Downregulated Apoptosis, and Upregulated the Expression of p-Akt/Akt and *p*-GSK3β/GSK3β *in vitro*

**FIGURE 1:**
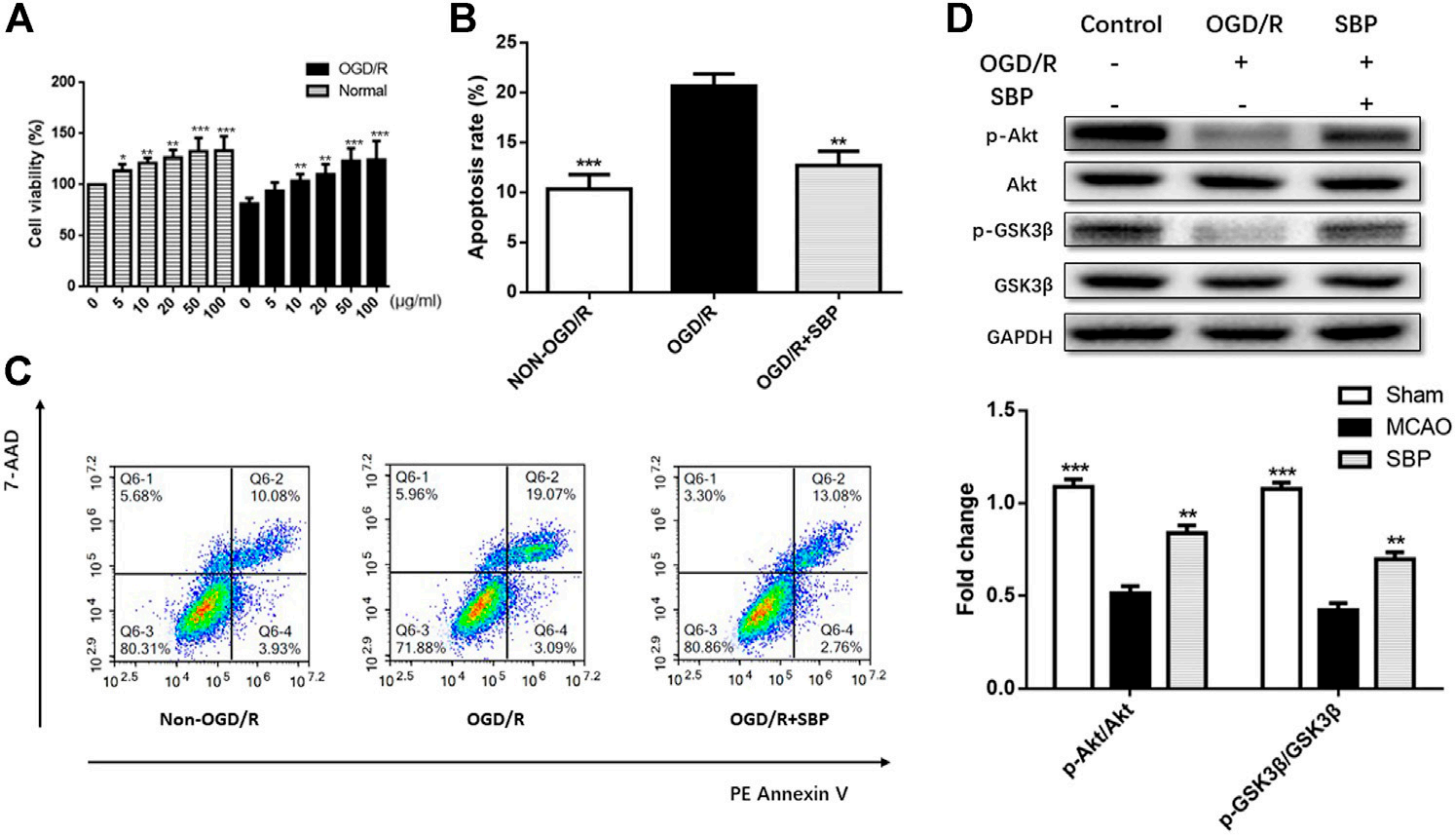
SBP enhanced cell viability and downregulated apoptosis, and upregulated the expression of p-Akt/Akt and p-GSK3β/GSK3β *in vitro*. **(A)** CCK8 assay for cell viability in CATH. a cells under OGD/R conditions with or without SBP. **(B, C)** The apoptosis of CATH. a was assessed by flow cytometry, and the results showed that OGD/R can enhance apoptosis, while the apoptosis was downregulated upon SBP treatment. **(D)** The protein levels of p-Akt/Akt and p-GSK3β/GSK3 were upregulated and activated by SBP. All data are means ± SEM, *n* = 3; ^*^ *p* < 0.05, ^**^ *p* < 0.01, and ^***^ *p* <.001 compared to OGD/R group.

The viability of CATH. a was detected by CCK8 assay. After 18 h co-treatment of SBP extract, the viability of CATH. a was enhanced after exposure to 10–100 μg/ml SBP (Figure 1A). Apoptosis of CATH. a was evaluated following the exposure of cells to 50 ug/mL of SBP. Flow cytometry confirmed that SBP reduced apoptosis of CATH. a. The ratio of apoptotic cells was proportional to the treatment time of SBP (Figures 1B,C). The results showed that SBP enhanced cell viability and downregulated apoptosis. We detected the protein expression of p-Akt and p-GSK3β by western blot in CATH. a, the results showed that compared to the OGD/R group, the expression of p-Akt and p-GSK3β was suppressed in CATH. a. The levels of p-Akt/Akt and p-GSK3β/GSK3β were elevated after treatment with SBP (Figure 1D).

### Shexiang Baoxin Pill Reduced Cerebral Infarction at Early Stage of Stroke in Rats 24 h After Ischemia/Reperfusion Brain Injury

**FIGURE 2:**
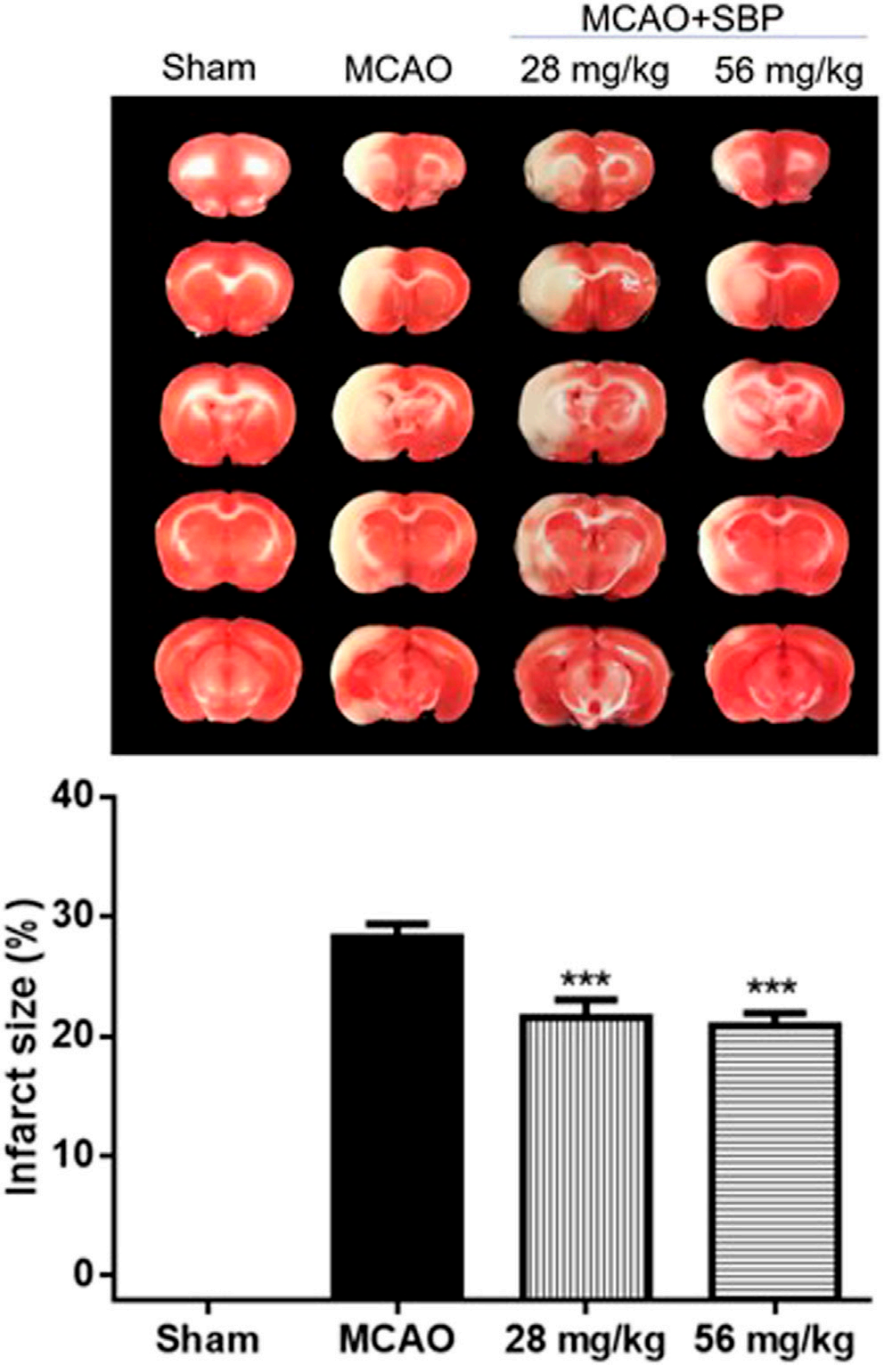
SBP reduces cerebral infarction at the early stage of stroke in rats 24 h after ischemia/reperfusion injury. Data are expressed as mean ± SEM, *n* = 8; ^***^ *p* < 0.001 compared to MCAO.

SBP (28 mg/kg or 56 mg/kg, p. o.) was administered at 1 h after ischemia, rats were killed at 24 h after reperfusion. Compared with MCAO rats, both 28 mg/kg SBP and 56 mg/kg SBP mg/kg treatment groups significantly reduced brain infarction (both *p* < 0.001). No significant difference was observed based on the current protocol between the different doses of SBP (Figure 2).

### Shexiang Baoxin Pill Improved Behavioural Effects on Locomotor, Cognitive Performance, and Anxiety in Rats 14 days After Ischemia-Reperfusion Brain Injury

**FIGURE 3:**
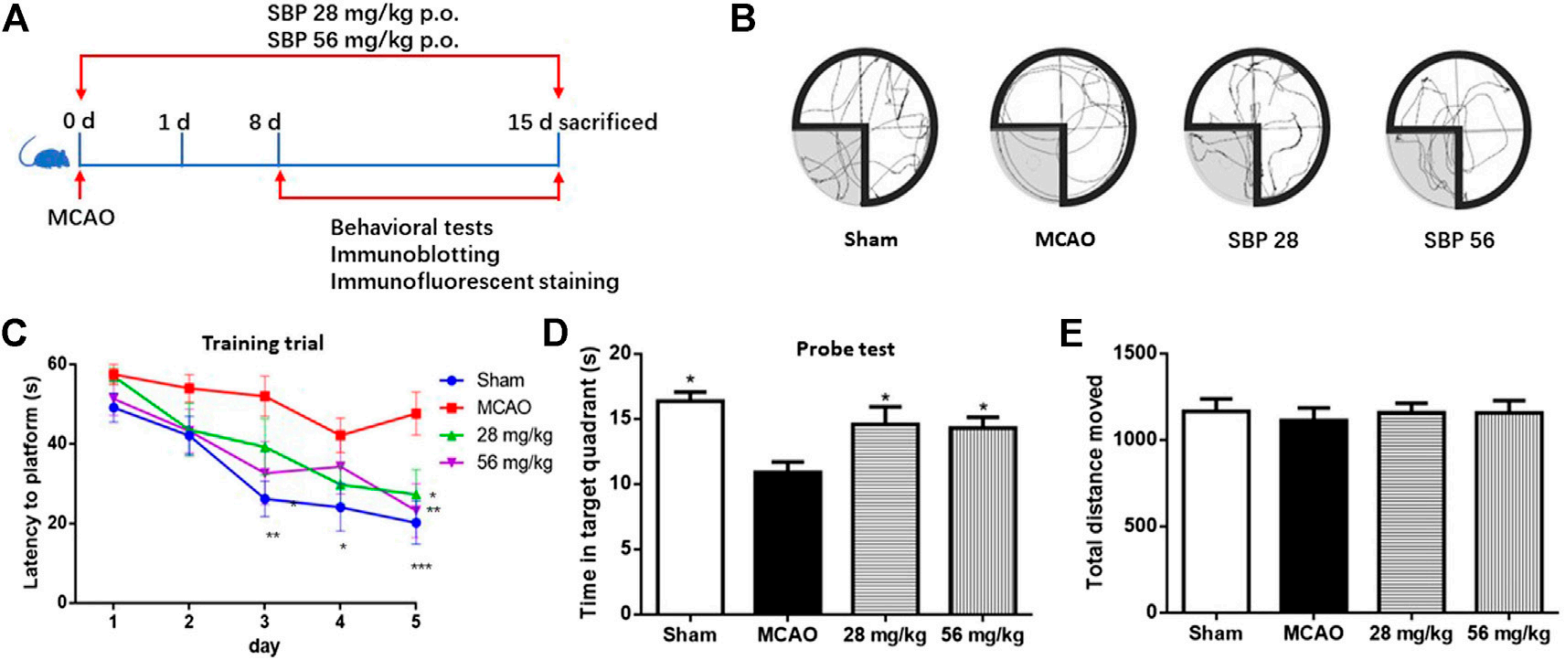
SBP Improved Behavioural Effects on Cognitive Performance in Rats 14 days after Ischemia-Reperfusion Brain Injury. Effects of SBP in MCAO model rats in the Morris water maze test. **(A)** Schematic representation of the experimental design. **(B)** representative swimming path. **(C)** Escape latency to the hidden platform in training trials. **(D)** time stay in the target quadrant. **(E)** total distance move. Data are expressed as mean ± SEM, *n* = 9; ^*^ *p* < 0.05, ^**^ *p* < 0.01, ^***^ *p* < 0.001 compared to MCAO.

**FIGURE 4:**
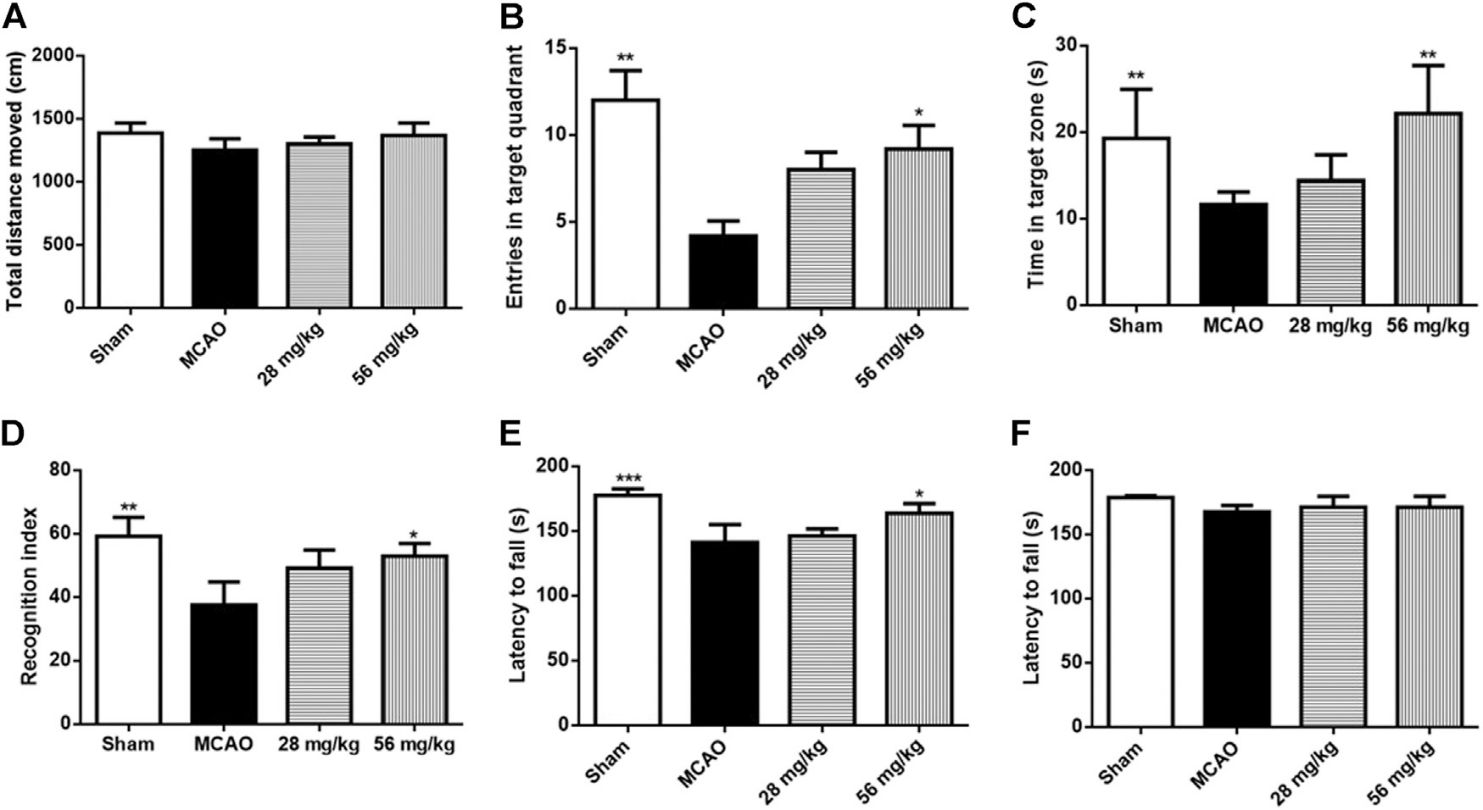
SBP Improved Behavioural Effects on Locomotor and Anxiety in Rats 14 days after Ischemia-Reperfusion Brain Injury. **(A)** representative open field test path **(B)** Entries in the target quadrant during the probe test. **(C)** Time stay in target quadrant during open field test. **(D)** Recognition index from noval object recognition. **(E)** Time to stay at rotarod at day 7. **(F)** Time to stay at rotarod at day 14. Data are expressed as mean ± SEM, *n* = 9: ^*^ *p* < 0.05, ^**^ *p* < 0.01, ^***^ *p* < 0.001 compared to MCAO.

SBP (28 mg/kg or 56 mg/kg) was administered before reperfusion for 14 days (Figure 3A). In the training trials of MWM, there was a significant effect over time [F (4, 32) = 18.96, *p* <.001] and also a significant effect for treatment [F (3, 24)]5.064, *p* = 0.007), no interaction effect of time and treatment was observed according to the present data [F (12, 96) = 0.883, *p* = 0.567]. Post-hoc comparisons showed that the latency of the MCAO group was significantly longer than that of vehicles on the 4 and 5th day (Figures 3B,C). Both SBP 28 and 56 groups markedly reduced the latency to the hidden platform on the 5th day. Higher dose SBP treatment (56 mg/kg) reduced the latency to the hidden platform on 3rd day. After SBP co-treatment, the MCAO-induced increase in escape latency was significantly improved (28 mg/kg vs. MCAO, *p* = 0.015; 56 mg/kg vs. MCAO, *p* = 0.002). There was no significant difference between the 28 mg/kg SBP group and 56 mg/kg SBP group in escape latency (*p* = 0.929). In the probe test of MWM, significant group effects were observed on the duration spent and the number of entries into the targeted quadrant, but not on distance moved (Figures 3D,E). There is no significant difference between groups in terms of total distance moved in the open field test (Figure 4A). The number of entries into the target quadrant was significantly greater in the 56 mg/kg SBP group compared with the MCAO group (*p* < 0.05) (Figures 4B,C). In novel object recognition and open filed tests, 58 mg/kg SBP treatment had better results compared with the MCAO group (*p* < 0.05 and *p* < 0.01, respectively) (Figure 4D). Higher dose SBP (56 mg/kg) restored locomotor function of stroke rats 7 days after ischemia (56 mg/kg vs. MCAO, *p* = 0.019; Figure 4E), no statistical difference between groups was found at day 14 (Figure 4F).

### Shexiang Baoxin Pill Upregulated the Expression of p-Akt, p-GSK3β, NMDAR1, PSD-95, AMPAR and Downregulated p-CAMKII in Rats 14 days After Ischemia/Reperfusion Injury

**FIGURE 5:**
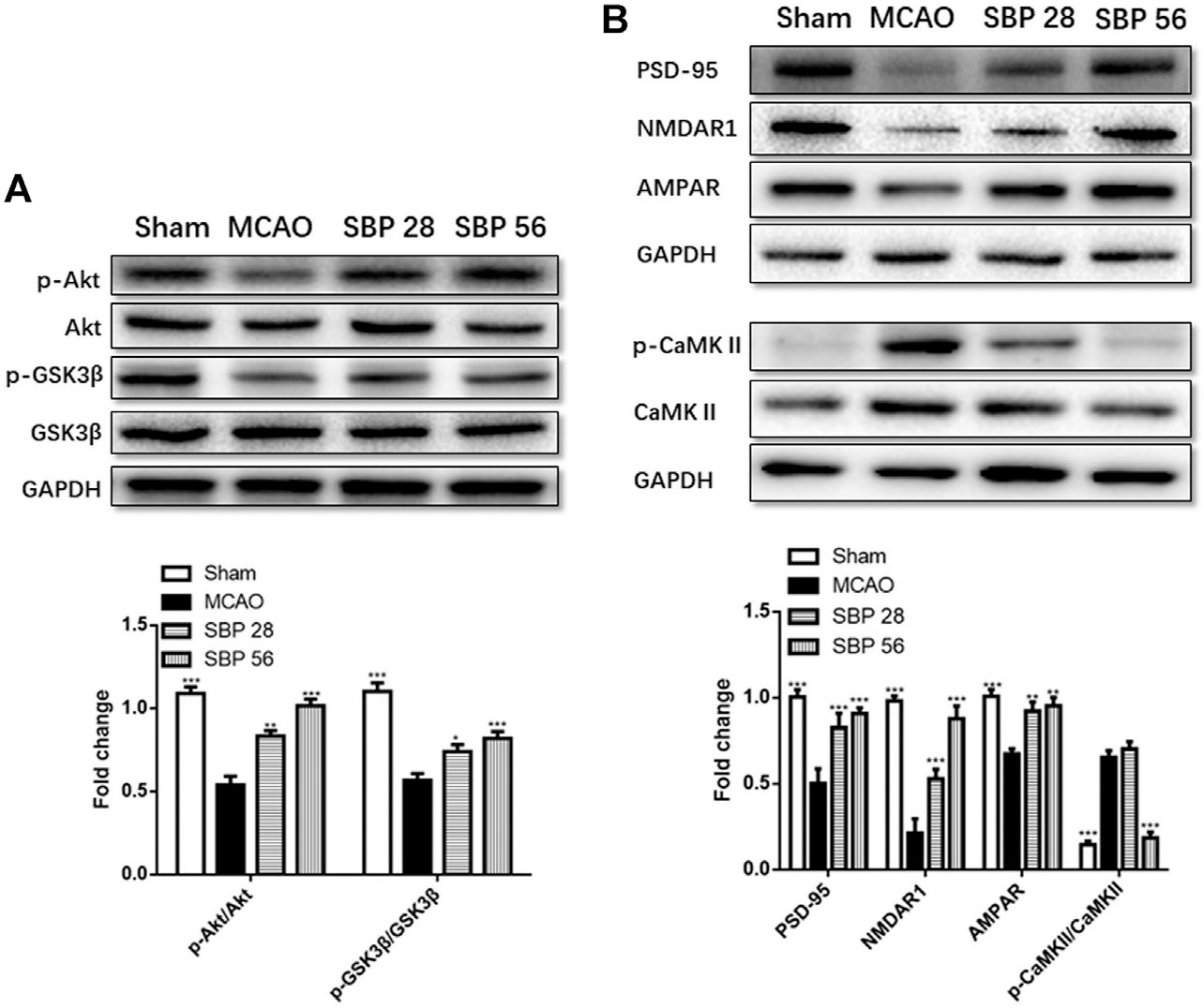
**(A)** Western blot analysis of p-Akt, Akt, p-GSK3β, and GSK3 in the ipsilateral side of the brain of rats at 14 d after MCAO (or sham surgery) operation. **(B)**Western blot analysis of PSD-95, NMDAR1, AMPAR, p-CaMKII, and CaMKII in the ipsilateral hippocampus of rats at 14 days after MCAO (or sham surgery) operation. Data are expressed as mean ± SEM, *n* = 5; ^*^ *p* < 0.05, ^**^ *p* < 0.01, and ^***^ *p* < 0.001.

**FIGURE 6:**
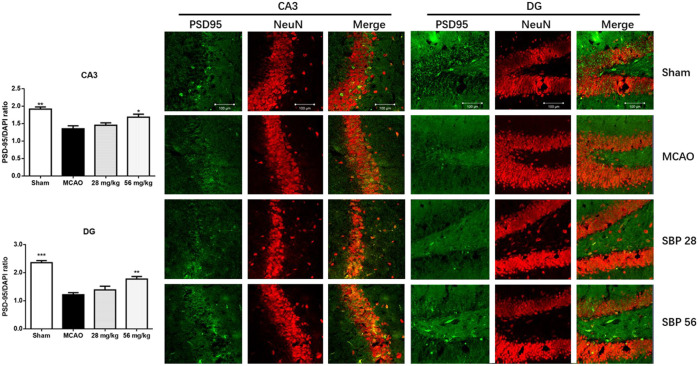
Immunofluorescence images on PSD-95 (green) and NeuN (red) in the cornu ammonis 3 (CA3) and dentate gyrus (DG) of hippocampal subregions of rats at 14 days after MCAO (or sham surgery) operation. Data are expressed as mean ± SEM, *n* = 3; ^*^ *p* < 0.05, ^**^ *p* < 0.01, ^***^ *p* < 0.001 compared to MCAO.

The expression of biomarkers in the rats was analyzed with western blot in the ipsilateral hippocampus of sham, MCAO, SBP 28 mg/kg, and SBP 56 mg/kg groups. p-Akt and p-GSK3β were both suppressed in MCAO rats after 14 days. Followed by SBP administration, the levels of p-Akt/Akt and p-GSK3β/GSK3β were increased [F (3, 32) = 57.00, *p* < 0.0001]. One-way ANOVA revealed significant effects of both SBP co-treatment groups on p-Akt/Akt (*p* < 0.001 for SBP 28 and SBP 56) and p-GSK3β/GSK3β (*p* = 0.021 for SBP 28 and *p* = 0.0007 for SBP 56) (Figure 5A). Similar results were found in CATH. a cell line. Besides, the MCAO rats had strikingly lower levels of NMDAR1, PSD-95, and AMPAR. Meanwhile, MCAO caused a dramatic overexpression of p-CaMKII. Both 28 and 56 mg/kg SBP co-treatment partially reversed all these changes compared to the MCAO rats (*p* < 0.01 for PSD-95, and *p* <.001 for NMDAR1, AMPAR, and p-CaMKII). No significant differences were found between different doses of SBP treatment (Figure 5B). Immunofluorescence was used to detect NeuN and PSD-95 markers in the dentate gyrus of the ipsilateral hippocampus, immunolocalization of PSD-95 in the dentate gyrus (DG) and cornu ammonis 3 (CA3) subregions was performed after 14 days following the operation. The DG and CA3 subregions in the sham group did not appear injured and instead showed a characteristic pattern of staining for NeuN, as well as abundant PSD-95 staining. Whereas in the MCAO group, changes in NeuN or PSD-95 immunostaining were not observed for the 14 days. Co-treatment with SBP, in particular with the higher dose (56 mg/kg) that was administered 14 days, prevented MCAO-induced PSD-95 decreasing (Figure 6).

## Discussion

The results of this study illustrated the therapeutic effects of SBP as a novel therapy in improving cognitive and locomotor impairment after ischemic stroke. A series of behavioral tests including the water maze test, locomotor ability test, and novel object recognition test showed that 14 days MCAO procedure significantly impaired spatial learning, memory, and locomotor ability. Also, MCAO markedly decreased entries and time into the central zone in the open field test, suggested that MCAO also induced anxiety-like behavior in rats. SBP in particularly higher dose reverse the anxiety-like behavior and prevent spatial learning, memory, and locomotor ability of MCAO rats.

It has been confirmed that stroke could result in cognitive impairment and the prevalence of post-stroke cognitive impairment in stroke survivors is high worldwide (Pendlebury and Rothwell, 2009). So far, no unequivocally efficacious management could be used for post-stroke cognitive impairment (Gorelick et al., 2011). Some agents used in Alzheimer’s disease such as cholinesterase inhibitors (donepezil, galantamine, and rivastigmine) and memantine have shown modest-to-no effects in clinical trials. However, the results from different studies were inconsistent and the benefit of these agents on post-stroke cognitive impairment is still uncertain. Although Alzheimer’s disease and post-stroke cognitive impairment are not the same, there is evidence suggesting that post-stroke cognitive impairment is involved in the pathogenesis of Alzheimer’s disease. Approximately 50% of dementias are attributed to both vascular cognitive impairment and Alzheimer’s disease. The clinical study suggested that the pathogenesis of Alzheimer’s disease makes contributions to more than 30% of demented cases after stroke. The latest study found that SBP might ameliorate the cognitive impairment in APP/PS1 transgenic mice via inhibition of Aβ fibril formation and suppression of secretions of cytokines (Tsoi et al., 2019). Hu et al. found that the expression of Bax was reduced and Bcl-2 was increased in SBP-treated APP/PS1 mice, also suggested that SBP exerts neuroprotective effects via an anti-apoptotic pathway (Tsoi et al., 2019).

The PI3K/Akt signaling pathway involves in many pathology processes, including apoptosis, proliferation, and inflammation (Peltier et al., 2007). Reducing apoptosis in the ischemic penumbra, where cells are potentially rescued after stroke plays a vital role in the prognosis. GSK3β is downstream of Akt, these proteins are combined and phosphorylated to regulate cellular metabolism. Previous studies suggested that the activation of the PI3K/Akt signaling pathway is important for neuroprotection against ischemia-induced apoptosis (Yao et al., 2012). In this study, the results from *in vitro* experiments revealed that in the OGD/R-induced CATH. a cell line, the member of PI3K/Akt signaling pathway was suppressed and similar results were found in the brain tissue of MCAO model rats after co-treatment with SBP. The neuroprotective effects of SBP might be attributed to its anti-apoptosis by activating the PI3K/Akt signaling pathway. This result may explain the clinical utility of SBP to a certain extent and will contribute to the subsequent studies.

Neuroplasticity contributes to brain repair, thereby improving post-stroke outcomes. The activation of NMDARs is crucial for dendritic plasticity and synaptogenesis (Schwarzbach et al., 2006). Mediated by NMDARs, Ca^2+^ influx plays a vital role in neuroplasticity. Meanwhile, the damage of synaptic function could lead to learning and memory deficits and has been confirmed by long-term potentiation (LTP) impairment (Walsh et al., 2002). During LTP *in vitro* PSD-95 controls activity-dependent AMPA receptor incorporation at synapses (Ehrlich and Malinow, 2004). Current data present after MCAO surgery, a significant reduction in the hippocampal expression of PSD-95 and the loss of PSD-95 correlated with a reduction in cognitive function, which could explain the neurological deficits after the initial ischemic stroke. We also found that the expression of NMDA and CAMKII were decreased on the 14th day after ischemic stroke. The overexpression of PSD-95 could mimic some key functions of LTP and experience-driven synaptic plasticity, including the enhancement of AMPA receptor-mediated transmission and translocation of GluR1 into synapses (Ehrlich and Malinow, 2004). In this study, we found that the expression of NMDAR1 and PSD-95 in the hippocampus was significantly decreased by MCAO. Besides, MCAO as well caused a dramatic overexpression of p-CaMKII, and SBP reversed the changes. Accordingly, the underlying neuroplasticity mechanisms of SBP might be associated with its activation of NMDAR1 and PSD-95.

Some limitations should be noticed in this study. First, the damaged synaptic function of MCAO-induced post-stroke cognitive impairment was not confirmed by LTP impairment through the electrophysiological test. Second, the study time point may not long enough, we only investigated 14 days treatment effect, other time points such as 28 days or longer might reveal more precise information in terms of the process post-stroke cognitive impairment.

## Conclusion

Current data suggested that SBP plays an anti-apoptotic role in the OGD/R-induced neuronal cells. In ischemic stroke induced cognitive impairment rats, SBP ameliorated learning and spatial memory impairment and improved locomotor function. The underlying mechanisms of SBP might be attributed to its neuroprotective anti-apoptosis effect as well as promoting hippocampal synaptic neuroplasticity in MCAO rats.

## Data Availability

The raw data supporting the conclusions of this article will be made available by the authors, without undue reservation.
