# Supplementary material for: Shexiang Baoxin Pill, a Proprietary Multi-Constituent Chinese Medicine, Prevents Locomotor and Cognitive Impairment Caused by Brain Ischemia and Reperfusion Injury in Rats: A Potential Therapy for Neuropsychiatric Sequelae of Stroke
Source: Front Pharmacol. 2021 Apr 27;12:665456. doi: 10.3389/fphar.2021.665456 (PMC8111446; doi:10.3389/fphar.2021.665456)
Supplement: Supplementary file 1 [file Table1.DOCX]

**Table 1.** Individual medicinal materials of the SBP

| **Material name** | **Ratio** | **Full scientific name** | **Major pharmacologically active constituents** |
| --- | --- | --- | --- |
| Artificial Mouchus | 6% | The dried preputial secretion of *Moschus berezovskii Flerov,* *Moschus sifanicus Przewalski,* or *Moschus moschiferus Linnaeus* | Muscone, testosterone |
| Calculus Bovis Artifactus | 27% | *Panax ginseng* C.A. Mey., root | Cholic acid, deoxycholic acid, ursodeoxycholic acid, chenodeoxycholic acid, bilirubin,cholesterol |
| Radix Ginseng | 24% | *Cinnamomum cassia* (L.) J. Presl., bark | Ginsenoside Ra1/2, Ginsenoside Rb1/2/3, Ginsenoside Re |
| Venenum Bufonis | 4% | The dried secretion of *Bufo bufo gargarizans Cantor* or *Bufo melanostictus Schneider* | Cinobufagin, resibufogenin, resibufagin, gamabufotalin, bufalin, 1β-hydroxylbufalin, arenobufagin, bufotalin, telocinobufagin, telibufagin |
| Cortex Cinnamomi | 8% | *Liquidambar orientalis* Mill., resin | Cinnamic acid, cinnamaldehyde |
| Styrax | 4% | The dried gall-stone of *Bos taurus domesticus Gmelin* | Benzyl benzoate |
| Borneolum Syntheticum | 19% | *Borneolum Syntheticum* or *Dryobalanops aromatica* C.F. Gaertn, resin | Borneol, isoborneol |
