# Supplementary figures and images for: Shexiang Baoxin Pill, a Proprietary Multi-Constituent Chinese Medicine, Prevents Locomotor and Cognitive Impairment Caused by Brain Ischemia and Reperfusion Injury in Rats: A Potential Therapy for Neuropsychiatric Sequelae of Stroke
Source: Front Pharmacol. 2021 Apr 27;12:665456. doi: 10.3389/fphar.2021.665456 (PMC8111446; doi:10.3389/fphar.2021.665456)

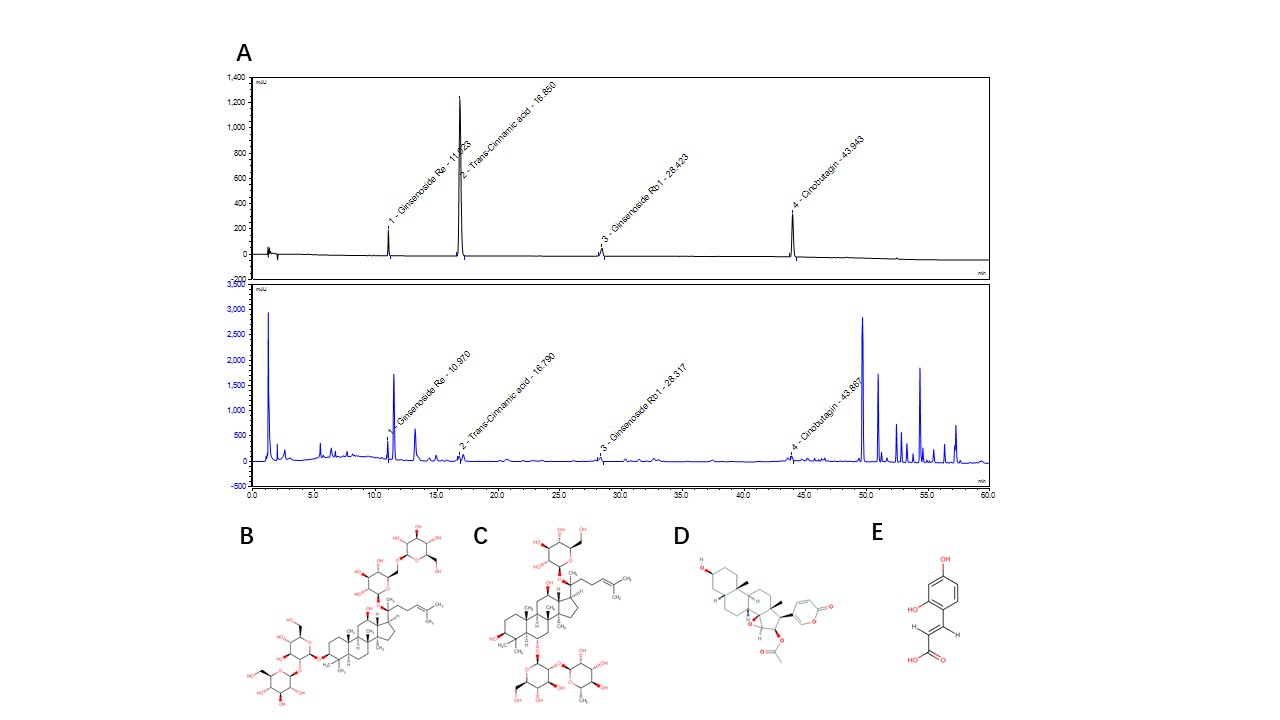

Supplement: Supplementary file 2 [file Image1.JPEG]
